# Effectiveness of a Transdiagnostic Emotion‐Focused Treatment in Clinical Care: A Sequential Single‐Case Experimental Design

**DOI:** 10.1002/ejp.70310

**Published:** 2026-06-13

**Authors:** Hedvig Zetterberg, Xiang Zhao, Sofia Bergbom, Rebecca Lennartsson, Ida Flink, Steven Linton, Katja Boersma

**Affiliations:** ^1^ Department of Education, Psychology and Social Work Mid Sweden University Östersund Sweden; ^2^ Center for Health and Medical Psychology, School of Behavioral, Social, and Legal Sciences Örebro University Örebro Sweden; ^3^ Department of Government and Society College of Humanities and Social Sciences, United Arab Emirates University AI Ain UAE; ^4^ University Health Care Research Center, Faculty of Medicine and Health Örebro University Örebro Sweden; ^5^ Centre for Psychiatry Research, Department of Clinical Neuroscience Karolinska Institutet, and Stockholm Health Care Services Stockholm Sweden

**Keywords:** chronic pain, cognitive‐behavioural therapy, depression, emotion regulation, exposure

## Abstract

**Background:**

Chronic pain frequently co‐occurs with emotional problems, highlighting the need for integrated treatment approaches. Previously, we evaluated a hybrid emotion‐focused treatment with positive results. However, there is a dire need for studies evaluating treatments in ‘real world’ clinical contexts. Therefore, this study evaluated the effectiveness of this treatment in regular clinical care.

**Methods:**

Using a sequential single‐case experimental AB design with randomized baseline lengths (4, 5 or 6 weeks), pain, emotional problems and cognitive behavioural factors were assessed weekly across a 20‐week study period. Additional outcome measurements were collected at pre‐, post‐ and 12‐month follow‐up. Patients from primary and secondary care (final *n* = 31) with chronic pain and anxiety/depression were recruited, and their psychologists delivered the treatment. Individual effects were evaluated using non‐overlap of all pairs and aggregated effects via multilevel shift models. Acceptability, adherence and fidelity were summarized, and responders and non‐responders compared using group‐level analyses.

**Results:**

Most (87%) demonstrated a treatment response on ≥ 1 outcome, and over half (55%) on ≥ 3 outcomes. One‐third were classified as broad responders. Aggregated analysis showed a significant effect on pain interference. The treatment was viewed as credible and acceptable, with greater satisfaction among responders. Non‐responders showed more variability in attendance and lower progression through later treatment phases involving exposure.

**Conclusion:**

The findings support the feasibility and potential effectiveness of the hybrid emotion‐focused treatment for patients in routine clinical care. The treatment benefits most patients to some extent and a smaller subset of patients to a clinically significant extent.

**Significance Statement:**

This pragmatic trial evaluates effectiveness in a clinically representative patient sample reflecting the complexity of routine care, including pain and psychiatric comorbidity, sociodemographic diversity and health care utilization. The hybrid emotion‐focused treatment represents one of several emerging approaches targeting pain–emotion connections. These findings extend results from a prior RCT and highlight the importance of evaluating interventions under real‐world clinical conditions.

## 1 Introduction

Chronic pain is often accompanied by anxiety and depression and this comorbidity presents treatment challenges (Asmundson and Katz 2009; Bair et al. 2003; De La Rosa et al. 2024). Specifically, depressive and anxiety symptoms are associated with reduced quality of life, poorer functioning and more pain, and negatively predict treatment outcomes (Gerdle et al. 2019; Tseli et al. 2020). Traditional cognitive behavioural pain management models frequently fail to sufficiently address emotional problems and lead to suboptimal patient outcomes (Gerdle et al. 2019; Michal et al. 2025; Morley et al. 2008; Williams et al. 2020). In addition, the allocation of patient‐to‐treatment is often based on the identification of a ‘primary’ problem area (e.g., either pain or mental health problems), complicating the assignment with appropriate treatment for patients who suffer from both.

From a transdiagnostic perspective, pain and emotional problems may be addressed jointly, with common and integrated treatment targets (Linton 2013; Sharp and Harvey 2001). In a series of studies (Boersma et al. 2019; Linton and Fruzzetti 2014; Södermark et al. 2020), we developed and evaluated a transdiagnostic emotion‐focused exposure treatment—coined the hybrid emotion‐focused treatment—which integrates exposure methods based on the fear‐avoidance model (Vlaeyen et al. 2016) with emotion‐regulation methods from Dialectical Behaviour Therapy (DBT) (Koerner 2012). The target population is individuals suffering from chronic pain and emotional problems, specifically persistent (> 3 months) musculoskeletal pain accompanied by substantial daily interference and high levels of emotional distress (Bergbom et al. 2025). In our randomized controlled efficacy trial, we found improvements in pain catastrophizing and pain interference post‐treatment, and in depression and pain interference at long‐term follow‐up, compared to a control group receiving guided Internet‐delivered cognitive‐behavioural therapy (iCBT) (Boersma et al. 2019). Improvements in pain‐related regulation and emotion regulation mediated the effects, supporting the transdiagnostic theoretical underpinnings of the treatment model (Södermark et al. 2020). Hence, this hybrid emotion‐focused treatment has the potential to enhance the outcome of chronic pain treatments, but the effects when rolled out in regular clinical care need further exploration.

This pragmatic trial builds on the promising results of our initial studies. Specifically, we seek to explore treatment effectiveness in regular clinical care. Pragmatic trials imply both a broadening of the target population and delivery by clinicians in ‘real world’ clinical contexts (Kazdin 2023). We employ a combined effectiveness‐implementation study design, focusing on testing treatment effects on relevant outcomes while simultaneously observing and gathering information for a process evaluation of treatment acceptability, adherence and fidelity (Bernet et al. 2013; Curran et al. 2012). We use a sequential single‐case experimental (SCED) design, which reveals detailed information at the individual patient level, and evaluation of effects aggregated across patients (Van den Noortgate and Onghena 2008; Vlaeyen et al. 2022). Rather than making inferences from the group to the individual, SCED provides evaluation for each participant and is thus particularly suitable for pragmatic clinical trials.

Specifically, we investigated the effects of the hybrid emotion focused treatment on pain problems (pain severity, pain interference), emotional problems (symptoms of depression, anxiety) and cognitive behavioural factors (worry, avoidance, symptom catastrophizing). In addition, patient and therapist evaluations of effects were assessed. Further, process evaluation included acceptability, treatment adherence and fidelity.

## 2 Material and Methods

### 2.1 Overview of Study Design

This study used a sequential single‐case experimental AB design (Michiels and Onghena 2019; Vlaeyen et al. 2022) with randomized baseline lengths of 4, 5 or 6 weeks, assessing outcomes weekly across the study period (20 weeks, including a baseline phase (A) and a treatment phase (B)). The randomized design reduces confounding between intervention effects and within‐person time‐related processes, thereby increasing internal validity (Heyvaert and Onghena 2014). Additional standardized outcome measurements were collected at pre‐treatment, post‐treatment and 12‐month follow‐up. Treatment providers (psychologists) filled out therapist reports post sessions. Participants were recruited between October 1, 2021 and January 1, 2023. The study was approved by the Swedish Ethical Review Authority (EPN Uppsala D‐No 2020–06083; 1–2) and conducted in accordance with the Declaration of Helsinki. Written informed consent was obtained from all participants. Reporting has been guided by the SCRIBE guidelines (Tate et al. 2016). The study was preregistered at ClinicalTrials.gov, ID: NCT05082922.

### 2.2 Recruitment and Sample

Inclusion criteria were as follows:

1. Chronic pain, i.e., pain problems > 3 months.
2. Functional impairment operationalized as either a score > 20 on items 21–24 of the Örebro Musculoskeletal Pain Questionnaire (ÖMPSQ) (Linton and Boersma 2003) or a score > 3 on item 2 from the pain interference subscale from the Multidimensional Pain Inventory, Swedish version (MPI‐S) (Bergström et al. 1998; Kerns et al. 1985).
3. Emotional problems, operationalized as > 11 points on either subscale of the Hospital Anxiety and Depression Scale (HADS) (Bjelland et al. 2002; Zigmond and Snaith 1983; Brehaut et al. 2020).

Exclusion criteria included insufficient mastery of the Swedish language, severe psychiatric problems requiring immediate alternative treatment, ongoing psychological treatment and recent initiation (< 3 months) of psychopharmacological treatment for anxiety or depression.

Participants were recruited by psychologists employed at participating primary and specialized pain care units across Sweden. Eligible patients seeking regular care were first identified by unit referral groups, psychologists or other health care professionals (e.g., nurses, physiotherapists or physicians). Potential participants received thereafter verbal and written study information from the psychologist and completed a screening form assessing inclusion criteria. Diagnostic assessment was conducted by the psychologist with individuals who fulfilled initial inclusion criteria to ensure eligibility. Following informed consent, an independent study coordinator randomized participants to a 4‐, 5‐ or 6‐week baseline, and contacted them to fill out the baseline questionnaire and start the baseline phase. After completion, the study coordinator coordinated treatment starts with the treating psychologist.

**FIGURE 1:**
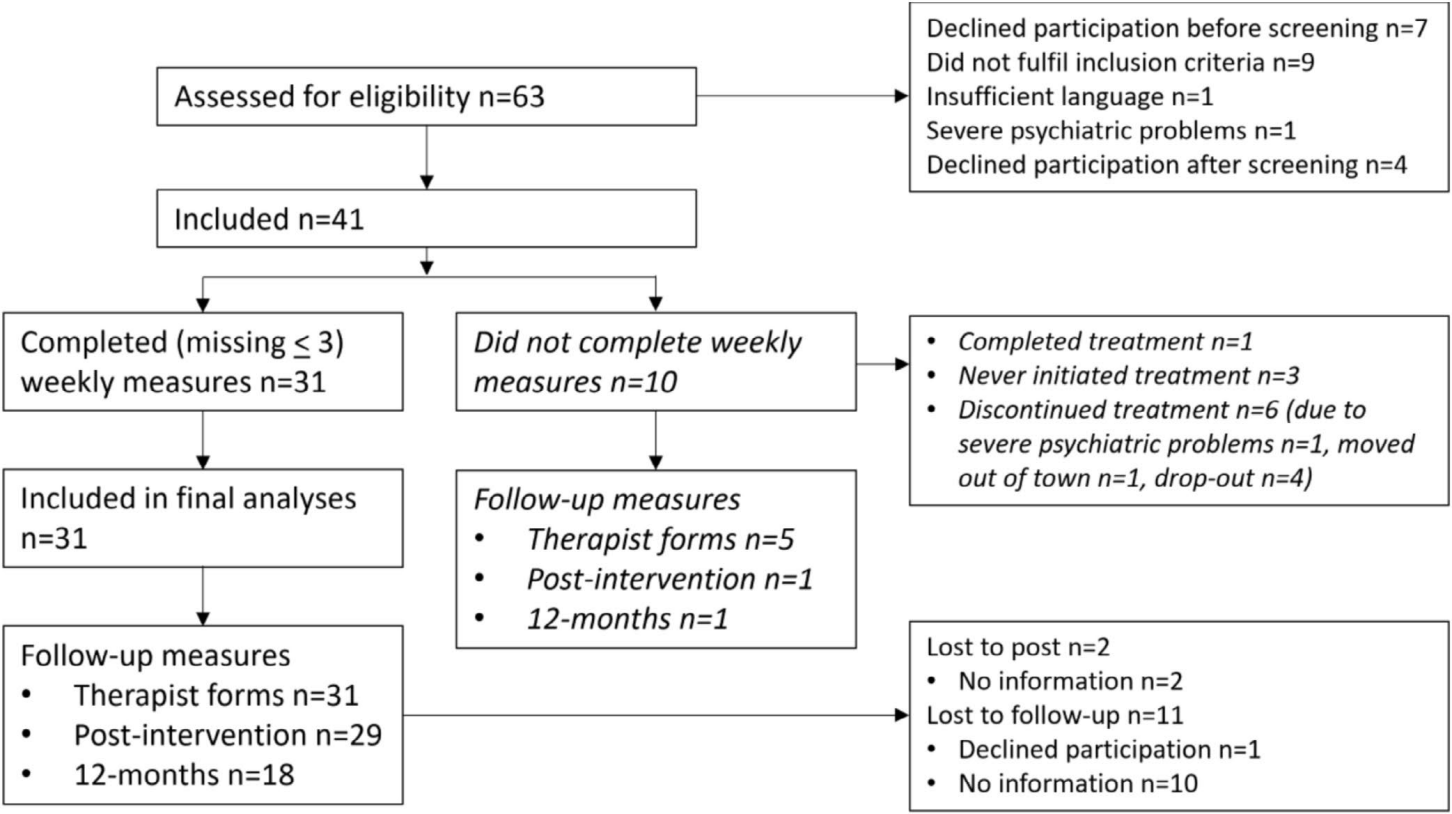
Flowchart of study participation and available data.

Figure 1 shows a flowchart of study participation. A total of 63 patients were assessed for eligibility, and an initial 41 participants were recruited. In an intention to treat approach, participants were asked to continue their study participation and provide data regardless of level of treatment adherence. To allow visual and statistical analyses of SCED data, a criterion of > 17 out of 20 data points on the weekly diary was set for inclusion in the study sample. Ten participants were lost to analysis because of insufficient data. Among these, three never initiated treatment, one participant completed treatment, and six discontinued treatment for various reasons (Figure 1). The participants lost to analysis did not differ from the final sample in baseline characteristics (see Table S1). In conclusion, 31 participants provided sufficient weekly diary data and made up the final study sample.

### 2.3 Therapists

All therapists were study trained. For therapists not previously trained in the method, training was delivered in two formats: (a) a designated 5‐week university course during the spring term of 2021 (7.5 credits, accredited by the Swedish Psychological Association) or (b) an online course with accompanying workshops during spring 2022 (four workshop days and 10 h of pre‐recorded lectures). A digital learning platform was created for the project, providing written materials, video demonstrations and recorded lectures.

Therapists (licensed psychologists with basic CBT training) were recruited through snowball sampling and advertisements in relevant Facebook groups. All therapists were offered group supervision every 6 weeks during the treatment period. In total, 43 psychologists received training and 16 chose to serve as therapists in the study. The participating therapists were employed at 14 different care sites across mid‐ and southern Sweden. The number of study participants treated per therapist ranged from 1 to 8 (median = 2). Care settings included specialist pain clinics in larger cities (population > 500,000) and primary care clinics in smaller towns (population < 50,000). Overall, 58% (*n* = 18) of participants were treated in specialist care and 42% (*n* = 13) in primary care.

### 2.4 Hybrid Emotion‐Focused Treatment

The hybrid emotion‐focused treatment aims to address shared underlying processes such as emotion dysregulation and avoidance, hypothesized to maintain comorbid pain and emotional problems (Bergbom et al. 2025; Linton 2013). The treatment is organized in 5 stages (I: Building a working relationship, soothing distress and developing relevant goals, II: Building skills to afford exposure, III: Exposure for avoided movement and emotions, IV: Building skills for emotion‐provoking social situations, V: Continuing progress, maintaining and refining), to provide a systematic approach while allowing for individualized tailoring (Table S2 provides an overview of the treatment). Each stage includes clearly outlined aims and suggested methods (Linton and Klein‐Strandberg 2020). A target of 10–15 sessions over a period of approximately 15 weeks was set.

### 2.5 Data Collection and Measures

Data were collected using self‐report questionnaires administered online via RedCap. Baseline demographic data included age, sex, educational level, occupational status, symptom duration, pain locations, sick leave and health care visits during the previous year. Weekly diary data were collected throughout the 20‐week study period, covering both the baseline (A) and intervention (B) phases.

The weekly diary included measures across two outcome domains: pain problems and emotional problems, covering symptom levels as well as functional interference. Measures of mechanisms were based on the transdiagnostic model (Linton 2013; Södermark et al. 2020) and covered cognitive behavioural factors: worry, catastrophizing and avoidance.

In addition, supplementary measurements using standardized questionnaires for emotional and pain‐related problems, work functioning and general health (described in Appendix S1) were administered at pre‐ and post‐treatment and at 12‐month follow‐up. Swedish versions of all questionnaires were used. Participants did not receive any compensation for completing the questionnaires. Therapists' treatment fidelity and patient treatment adherence were assessed using therapists‐reported session checklists, completed after each session. At the post‐treatment assessment, participants were also asked to report adverse events, using an open‐ended question.

### 2.6 Treatment Effects

#### 2.6.1 Weekly Diary

##### 2.6.1.1 Pain Problems

###### 2.6.1.1.1 Pain Severity

It was assessed using one item from MPI‐S (Bergström et al. 1998): ‘On the average, how severe has your pain been during the last week?’ rated from 0 = not at all severe to 6 = extremely severe.

###### 2.6.1.1.2 Pain Interference

It was assessed using two items from MPI‐S (Bergström et al. 1998). The first item assessed pain interference in general: ‘In general, how much does your pain problems interfere with your day‐to‐day activities?’ rated from 0 = no interference to 6 = extreme interference. The second item assessed activity limitation specifically: ‘How much do you limit your activities in order to prevent your pain from getting worse?’ rated from 0 = not at all to 6 = to a high degree.

##### 2.6.1.2 Emotional Problems

###### 2.6.1.2.1 Depressive Symptoms

They were assessed using the Patient Health Questionnaire 9‐item depression module (PHQ‐9), which has acceptable reliability and validity (Hansson et al. 2009; Kroenke et al. 2001). Patients rated symptom frequency during the past week (adapted from the original 2 weeks for the purpose of this study) on a 4‐point scale; sum scores ranged from 0 to 27. Cronbach's alpha in the current study was 0.82.

###### 2.6.1.2.2 Anxiety Symptoms

They were assessed using the Generalized Anxiety Disorder 7‐item scale (GAD‐7), which has good reliability and validity (Spitzer et al. 2006). Like the PHQ‐9, patients rated symptom frequency during the past week on a 4‐point scale; sum scores range from 0 to 21. Cronbach's alpha in the current study was 0.91.

###### 2.6.1.2.3 Emotional Interference

It was assessed using a shared item from the PHQ‐9 and GAD‐7: ‘If you checked any problems, how difficult have they made it for you to do your work, take care of things at home, or get along with other people?’ rated 0 = not difficult at all, 1 = somewhat difficult, 2 = very difficult, 3 = extremely difficult.

##### 2.6.1.3 Cognitive Behavioural Factors

###### 2.6.1.3.1 Worry

It was assessed using a study specific item: ‘During the last week, how often have you been worried about your future health?’ rated 0 = never, 1 = rarely, 2 = from time to time, 3 = often, 4 = all the time.

###### 2.6.1.3.2 Avoidance (Behavioural)

It was measured with a study specific item: ‘Due to your health problems: How often have you refrained from activities during the last week?’ rated 0 = never, 1 = rarely, 2 = from time to time, 3 = often, 4 = all the time.

###### 2.6.1.3.3 Avoidance (Cognitive)

It was assessed using a modified item from the Psychological Inflexibility in Pain questionnaire (Wicksell et al. 2010): ‘During the last week, how often have you been ruled by thoughts like “I don't have the energy”, “I am not well enough”, “I don't dare”, “I have too much pain” or “I feel too bad”’, rated 0 = never, 1 = rarely, 2 = from time to time, 3 = often, 4 = all the time.

###### 2.6.1.3.4 Catastrophizing

It was assessed using the Symptom Catastrophizing Scale (SCS) (Moore et al. 2018). The SCS was developed from the Pain Catastrophizing Scale and validated among patients with major depressive disorder (Moore et al. 2018). The SCS includes 7 items regarding thoughts and feelings related to participants' somatic or mental health condition, e.g., ‘I become afraid that my condition will get worse’ and ‘My symptoms are awful and I feel that they overwhelm me’. In the current study, a 5‐point scale was used, providing a sum scores range of 0–28. Cronbach's alpha in the current study was 0.90.

#### 2.6.2 Patient and Therapist Evaluation of Improvement

Patients rated their experiences of global improvement after treatment using two items: ‘Has your ability to cope with your symptoms been influenced by the treatment?’ and ‘Has your well‐being been influenced by the treatment?’ Items were rated from 0 = not improved at all to 5 = greatly improved and averaged to produce a total score for global improvement. A cut‐off at mid‐scale of ≥ 2.5 was used as a criterion signalling a positive patient evaluation of effect.

Therapists evaluated to what degree treatment targets were achieved at the end of treatment using a single item: To what extent do you believe the following goals have been addressed or achieved within the treatment: ‘Achieved personalized prioritized goals’. The item was rated on a 4‐point scale: 1 = deterioration, 2 = unchanged, 3 = positive and 4 = very positive. A cut‐off based on response descriptors, where scores ≥ 3 indicated improvement, was used as a criterion signalling a positive therapist evaluation of effect.

### 2.7 Process Evaluation

#### 2.7.1 Treatment Acceptability

Three items from the Credibility/Expectancy Questionnaire (Devilly and Borkovec 2000) were assessed at baseline: ‘How logical does the therapy offered to you seem for your problems?’, ‘How successful do you think this treatment will be?’ and ‘How confident would you be in recommending this treatment to a friend?’. In this study, items were rated from 0 to 6 where higher scores indicated higher credibility and averaged to form a total score. Cronbach's alpha in the current study was 0.78.

Treatment satisfaction was assessed after the treatment with the following two items: ‘How pleased are you with the treatment?’ and ‘Would you recommend the treatment to someone with similar problems?’. Items were rated from 0 to 5 where higher scores indicated higher satisfaction and averaged to form a total score. Cronbach's alpha in the current study was 0.89.

#### 2.7.2 Treatment Adherence and Fidelity

Therapist self‐report session checklists were used to assess treatment adherence and treatment fidelity. For each session, therapists recorded a brief description of the session content, session date and duration, reasons for missed sessions and whether and what homework had been assigned. Therapists also indicated which components of the hybrid treatment had been included in the session (See Table S2). Homework content was reported in a corresponding manner. Dichotomized variables were created, coded 1 when key components of a treatment phase had been reported during a session (signifying whether the treatment phase had been addressed). In a similar way, dichotomized variables were created on whether sessions included homework and its content. Based on these data, the following variables were created:

1. A variable denoting which treatment phases were addressed for each participant during the treatment (reported as *n*, %).
2. A variable denoting the proportion of sessions within each treatment that address advanced phases (defined as phases focusing on exposure; ≥ Phase III) for each participant (reported as median, range).
3. A variable denoting the proportion of sessions in which therapists assigned homework (reported as median, range). Also, a dichotomized variable was created to indicate whether participants had received homework specifically focused on exposure (once or more, reported as *n*, %)

### 2.8 Analyses

Analyses were based on available data, and no data imputation was performed. Therapist session reports were available for all included participants, though there were missing data in *n* = 10 on therapist evaluation of treatment effect.

To analyse the treatment effect aggregated across participants, a multilevel shift model with randomization test was used (Verboon et al. 2022). This method was deemed suitable for the study's purpose of estimating inferential statistics and effect sizes at the study level (Verboon et al. 2022). The average standardized treatment effect (delta) was interpreted according to conventional benchmarks: 0.2 = small, 0.5 = medium and > 0.8 = large effect.

To analyse treatment effect on the individual level we used the non‐overlap of all pairs (NAP) (Parker and Vannest 2009). The NAP is a commonly used nonparametric effect size estimate for single‐case AB designs (Manolov and Moeyaert 2017). We used a NAP value ≥ 0.65 as a criterion for treatment response (signalling a moderate effect; Parker and Vannest 2009) and describe the NAP value for each weekly measure separately. Validation of effects was conducted via visual inspection of graphed data, taking into account trend, level and latency (Kratochwill et al. 2013).

Furthermore, an average NAP ≥ 0.65 across all weekly measures was calculated as a criterion for a broad treatment response and to classify overall ‘responders’ and ‘non‐responders’. To validate this classification, we further used the Wilcoxon signed rank test to assess within‐group effects on the standardized measures from pre to post and from pre to 12‐months follow‐up, for the subgroups classified as responders and non‐responders. NAP calculations and graphing of figures were performed using the ‘scda’ package in R (Verboon et al. 2022).

For acceptability, treatment adherence and fidelity outcomes, descriptive statistics were calculated. These outcomes were further related to treatment response by comparing responders and non‐responders using between‐group statistics. Between‐group differences were assessed using the Mann–Whitney *U* test for continuous variables and the chi‐square test (alternatively, Fisher exact test when the assumption of the chi‐square test is violated) for categorical variables. For within‐group statistics for secondary standardized measures, paired *t*‐tests were used and effect sizes were interpreted by Cohen's *d*: 0.2 = small, 0.5 = medium, 0.8 = large, 1.2 = very large.

## 3 Results

### 3.1 Participants

Table 1 presents demographic and clinical characteristics of the study sample. Participants were, on average, middle‐aged, and 42% were not working or studying. Long‐term sick leave was common; 48% reported sick leave exceeding 180 days during the past year. The majority of participants (71%) were women and approximately two‐thirds (65%) had Swedish nationality. All participants had musculoskeletal pain and 93.5% had multisite pain symptom burden (≥ 3 locations), based on pain sites neck, shoulders, upper back, lower back, arms/legs, stomach and head. Health care utilization during the past year was also high. Diagnostic interviews indicated that 82% met criteria for an anxiety or depressive disorder, reflecting substantial functional interference due to emotional problems in the majority of the sample.

**TABLE 1:** Participants' demographic and clinical characteristics at baseline.

	*n* = 31
Age, mean (SD)	38.9 (13.0)
Gender, women, *n* (%)	22 (71.0)
Nationality, Swedish, *n* (%)	20 (64.5)
Education, *n* (%)	
Middle school	2 (6.5)
Highschool/vocational	23 (74.2)
University	6 (19.4)
Occupational status, *n* (%)	
Working	14 (45.2)
Unemployed	7 (22.6)
Student	4 (12.9)
Non‐working (e.g., retirement pension)	6 (19.4)
Sick leave past year, *n* (%)	
0–14 days	11 (35.5)
15–180 days	5 (16.1)
181–365 days	15 (48.4)
Pain locations, *n* (%)	
Back, neck and/or shoulders	30 (96.8)
Legs and/or arms (and feet and hands)	25 (80.6)
Stomach	17 (54.8)
Head (e.g., headache, face, jaws, eyes)	28 (90.3)
Pain classification	
Musculoskeletal pain	31 (100.0)
Widespread pain (spine and legs/arms)	24 (77.4)
Multisite symptom burden ≥ 3 sites	29 (93.5)
Number of pain locations^a^, median (IQR)	6 (3)
Symptom duration > 1 year, *n* (%)	30 (96.8)
Health care visits, median (range) past year	
Physician	5 (0–> 10 visits)
Specialist/hospital	2 (0–> 10 visits)
Psychologist	4 (0–> 10 visits)
Physiotherapist	8 (0–> 10 visits)
Social worker	0 (0–> 10 visits)
Other (e.g., chiropractor, acupuncturist)	0 (0–> 10 visits)
Total number of visits > 2, *n* (%)	30 (96.8)
Screening measures, mean (SD)	*n* = 17
Function (OMPSQ, 0–40)	27.1 (7.0)
Pain interference (MPI, 0–6)	5.1 (0.5)
HADS anxiety (0–21)	14.1 (3.7)
HADS depression (0–21)	13.2 (2.9)
Psychiatric comorbidity (MINI), *n* (%)	*n* = 22
Not fulfilling disorder criteria	4 (18.2)
Major depressive disorder	13 (59.1)
Anxiety disorder	13 (59.1)
Comorbid depressive and anxiety disorder	8 (36.4)

Abbreviations: HADS, Hospital Anxiety and Depression Scale; MINI, Mini International Neuropsychiatric Interview 7.0.1; MPI, Multidimensional Pain Inventory item 2; OMPSQ, Orebro Musculoskeletal Pain Screening Questionnaire items 21–24.

^a^ Seven pain sites in total: neck, shoulders, upper back, lower back, arms/legs stomach and head.

### 3.2 Treatment Effects

Table 2 gives an overview of treatment effects aggregated across participants. The multilevel shift model with randomization test showed a significant treatment effect for the general pain interference item (*δ* = 0.34, *p* = 0.005).

**TABLE 2:** Multilevel shift model with randomization test for weekly measures, *n* = 31.

	Average standardized treatment effect (delta)	*p*
Pain problems		
1. Pain severity	0.17	0.43
2. Pain interference general	0.34	0.045*
3. Pain interference activity limitation	0.23	0.744
Emotional problems		
4. Depressive symptoms	0.16	0.167
5. Anxiety	0.07	0.387
6. Emotional interference	0.0	0.995
Cognitive behavioural factors		
7. Worry	0.04	0.260
8. Avoidance	0.08	0.999
9. Avoidance	0.24	0.761
10. Symptom catastrophizing	0.06	0.766

* *p*‐value significant at < 0.05.

Table 3 presents an overview of treatment effects on the weekly measures, using NAP. Among the 31 participants, 87% demonstrated a treatment response on ≥ 1 outcome measure, and over half (55%) showed a treatment response on ≥ 3 outcome measures. Four participants (13%) did not show a treatment response on any of the outcome measures. Example graphs of a responder and a non‐responder are provided in Figure S1. Using an average NAP ≥ 0.65 across all measures as a criterion for a broad and robust treatment response, 10 of the 31 participants (32%) could be classified as treatment responders and 21 (68%) as non‐responders. This classification converged with statistically significant improvements with large effect sizes on most standardized measures from pre‐treatment to post‐treatment and follow‐up (See Table S3).

**TABLE 3:** Non‐overlap of all pairs (NAP) for weekly measures, *n* = 31.

Id	Glob. Imp.	Goal Ach.	Averaged NAP	Pain problems NAP			Emotional problems NAP			Cognitive behavioural factors NAP			
				1. P_sev	2. P_int	3. P_int	4. Dep	5. Anx	6. Int	7. Wor	8. Avoid	9. Avoid	10. SCS
1	4	—	0.81	0.88	0.83	0.71	0.88	0.80	0.79	0.79	0.78	0.80	0.88
2	1	—	0.70	0.88	0.75	0.75	0.68	0.85	0.62	0.53	0.52	0.68	0.73
3	3.5	2	0.66	0.65	0.71	0.87	0.77	0.70	0.48	0.70	0.39	0.47	0.86
4	4.5	2	0.65	0.50	0.67	0.65	0.82	0.52	0.65	0.65	0.65	0.66	0.69
5	5	3	0.79	0.64	0.81	0.77	0.87	0.60	0.77	0.83	0.78	0.88	0.91
6	5	4	0.73	0.69	0.51	0.71	0.90	0.81	0.61	0.64	0.71	0.86	0.89
7	3.5	—	0.76	0.87	0.59	0.63	0.99	0.65	0.60	0.67	0.76	0.87	0.98
8	4	4	0.65	0.89	0.94	0.42	0.89	0.43	0.50	0.37	0.60	0.50	0.94
9	3	—	0.76	0.84	0.88	0.84	0.89	0.83	0.63	0.44	0.62	0.77	0.85
10	0.5	3	0.65	0.50	0.68	0.95	0.61	0.32	0.48	0.76	0.61	0.75	0.80
11	—	—	0.52	0.48	0.68	0.51	0.66	0.20	0.69	0.49	0.44	0.67	0.38
12	4.5	—	0.48	0.30	0.56	0.73	0.82	0.39	0.47	0.29	0.33	0.40	0.48
13	0	—	0.49	0.53	0.50	0.44	0.20	0.54	0.50	0.50	0.50	0.38	0.80
14	0	2	0.45	0.63	0.69	0.31	0.41	0.41	0.31	0.44	0.37	0.59	0.29
15	0.5	2	0.32	0.50	0.50	0.50	0.22	0.09	0.20	0.25	0.38	0.50	0.06
16	5	4	0.40	0.27	0.53	0.40	0.43	0.26	0.37	0.43	0.32	0.71	0.30
17	—	3	0.27	0.21	0.29	0.41	0.02	0.04	0.45	0.44	0.46	0.39	0.01
18	1.5	3	0.30	0.21	0.50	0.50	0.26	0.25	0.19	0.24	0.54	0.24	0.04
19	2	3	0.46	0.21	0.33	0.58	0.55	0.82	0.50	0.28	0.48	0.66	0.20
20	3.5	3	0.56	0.40	0.88	0.52	0.49	0.55	0.36	0.34	0.74	0.53	0.75
21	2	2	0.39	0.20	0.41	0.45	0.61	0.39	0.46	0.54	0.30	0.53	0.01
22	1	—	0.48	0.19	0.26	0.23	0.79	0.70	0.56	0.54	0.16	0.50	0.86
23	3.5	3	0.60	0.57	0.67	0.64	0.39	0.72	0.50	0.42	0.70	0.83	0.54
24	1.5	2	0.37	0.36	0.38	0.72	0.02	0.16	0.66	0.31	0.53	0.28	0.23
25	1	2	0.55	0.69	0.57	0.76	0.63	0.51	0.44	0.39	0.84	0.52	0.14
26	2.5	2	0.54	0.56	0.70	0.68	0.35	0.56	0.50	0.39	0.62	0.52	0.54
27	—	—	0.39	0.43	0.56	0.42	0.54	0.37	0.12	0.28	0.25	0.29	0.67
28	2	2	0.56	0.38	0.34	0.20	0.46	0.91	0.59	0.66	0.58	0.78	0.70
29	0	—	0.54	0.86	0.77	0.64	0.49	0.46	0.35	0.52	0.35	0.45	0.49
30	5	3	0.60	0.91	0.81	0.32	0.88	0.64	0.57	0.34	0.52	0.56	0.45
31	3	—	0.60	0.60	0.64	0.77	0.63	0.80	0.58	0.30	0.59	0.44	0.60

*Note:* NAP ≥ 0.65 moderate effect: boxes in grey. The upper ten rows display the participants that are categorized as treatment responders, and the lower 21 rows display the non‐responders. 1. P_sev = 1. Pain severity, 2. P_int = 2. Pain interference, 3. P_int = 3. Pain interference, 4. Dep = 4. Depressive symptoms, 5. Anx = 5. Anxiety, 6. Int = 6. Emotional interference, 7. Wor = 7. Worry, 8. Avoid = 8. Avoidance, 9. Avoid = 9. Avoidance, 10. SCS = 10. Symptom catastrophizing. Global improvement = Participant judgement of global improvement (0–5, score ≥ 2.5 marked in grey). Goal achievement = therapist's evaluation of whether patients personally relevant goals have been achieved Items rated from 1 to 4 where 1 = deterioration, 2 = unchanged, 3 = positive and 4 = very positive. Scores 3 and 4 marked in grey to signify improvement.

Table 3 also includes an overview of patients' self‐evaluation of global improvement and therapists' evaluation of relevant goal achievement. Half of the participants (*n* = 14) scored an average of > 2.5 (mid‐scale) on global improvement, indicating that half the sample had a positive evaluation of the treatment resulting in improved coping and well‐being. Among available data, therapists rated goal achievement as either positive or very positive for *n* = 11 out of *n* = 20 (55%) of the participants. A cross‐tabulation of patient and therapist ratings of improvement is provided in Table S4.

### 3.3 Process Evaluation

Table 4 provides an overview of treatment acceptability. On average, participants viewed the treatment as credible, with no significant differences in credibility between responders and non‐responders. However, while both groups were on average satisfied with treatment, responders reported higher satisfaction with less variability between participants.

**TABLE 4:** Treatment acceptability.

	Total sample, *n* = 31	Responders *n* = 10	Non‐responders *n* = 21	Between‐group statistics
Pre‐treatment				
Credibility (0–6)	3.7 (1.7–6.0)	4.0 (3.3–6.0)	3.8 (1.7–6.0)	*U* = 130.50, *z* = 1.09, *p* = 0.28

	*n* = 28	*n* = 10	*n* = 18	
Post‐treatment				
Satisfaction (0–5)	3.5 (0–5.0)	4.5 (3.0–5.0)	3.3 (0–5.0)	*U* = 141.50, *z* = 2.51, *p* = 0.01*

*Note:* Numbers are median (range).

* *p*‐value significant at < 0.05.

Table 5 provides an overview of treatment adherence and fidelity. On average, participants in the study sample completed 10 sessions out of the designated 10–15. The most common reasons for missed sessions were cancellations or no‐shows. Four of the 31 patients dropped out of treatment after attending two, four, six and nine sessions, respectively, due to unknown reasons (*n* = 1), stress and lack of time (*n* = 2) or illness (*n* = 1). No adverse events were reported. While no significant differences in treatment duration were observed between responders and non‐responders, there is a notable difference in variability in session attendance (responders, range 8–15; non‐responders, range 2–15).

**TABLE 5:** Adherence to treatment and treatment fidelity.

	Total sample, *n* = 31	Treatment responders, *n* = 10	Non‐responders, *n* = 21	Between‐group statistics
Session attendance				
Attended sessions, median (range)	10 (2–15)	11 (8–15)	10 (2–15)	*U* = 120.50, *z* = 0.66, *p* = 0.52
Missed sessions^a^, median (range)	4 (0–13)	3 (0–7)	4 (0–13)	*U* = 88.00, *z* = −0.73, *p* = 0.47
Session duration minutes, mean (SD)	56.2 (10.3)	50.0 (16.2)	60.5 (14.6)	*U* = 74.00, *z* = −1.31, *p* = 0.20

	*n* = 30	*n* = 10	*n* = 20	
Phases addressed, *n* (%)				
Phase I–II	7 (23)	0 (0)	7 (35)	Fishers' exact test, *p* = 0.07^#^
Phase I–III	4 (13)	1 (10)	3 (15)	
Phase I–IV	10 (30)	6 (60)	4 (20)	
Phase I–V	9 (30)	3 (30)	6 (30)	
Proportion of sessions addressing phases ≥ 3, median (range)	26 (0–71)	38 (15–50)	16 (0–71)	*U* = 144.50, *z* = 1.97, *p* = 0.05*
Homework				
Proportion of sessions including homework, median (range)	90 (50–100)	91 (80–100)	89 (50–100)	*U* = 125.50, *z* = 1.15, *p* = 0.27
Participants with > 1 occasion of homework with exposure content, *n* (%)	17 (57)	8 (80)	9 (45)	Fisher's exact test, *p* = 0.07^#^

^a^ Include cancelled sessions, no‐show sessions, drop‐out of treatment and missing session information.

* *p*‐value significant at < 0.05.

^#^ *p*‐value significant at < 0.10.

Regarding treatment fidelity, there was variability in the extent to which and to what degree treatment phases were addressed across participants. Notably, among non‐responders, a lower progression through treatment phases was evident. A relatively large proportion (35%, *n* = 7) did not progress beyond Phase II. Only 50% (*n* = 10) of non‐responders received treatments that addressed Phases IV and V while this was the case for 90% (*n* = 9) of responders. In concordance, the proportion of treatment focused on more advanced phases (i.e., ≥ Phase III) was significantly lower among non‐responders.

Across participants, therapists included homework in a large proportion (90%) of the sessions. Yet, in the degree to which homework contained exposure there was a tendency towards a difference between responders and non‐responders, with responders receiving homework assignments related to exposure (80%, *n* = 8) to a higher degree than those classified as non‐responders (45%, *n* = 9).

## 4 Discussion

This study aimed to implement and evaluate the effectiveness of a hybrid emotion‐focused treatment for patients with comorbid chronic pain and emotional problems in front line clinical care. A reach of 14 different care settings, at both primary and secondary level, across different parts of Sweden was achieved. The patient sample was characterized by high levels of comorbidity across a broad spectrum of pain and mental indicators, confirming that the intended clinical target group was reached, which is the aim of pragmatic trials (Kazdin 2023). Included patients had a demographic profile of relatively low educational attainment, substantial sick leave, high health care utilization and a high proportion of immigrant status—indicators of complex and comorbid problems observed in clinical care (Gerdle et al. 2019).

At the individual level, most patients showed a treatment response on at least one outcome, over half on three or more outcomes, and approximately one‐third showed a more broad and robust treatment response across outcomes. Patient ratings of global improvement and therapist evaluations of outcomes were generally consistent with these findings. When including patients' and therapists' judgements, a positive evaluation of the treatment was observed in about half of the sample. While there is an overlap between outcome‐based and subjective evaluations, these perspectives do not always align—a phenomenon previously reported in the literature (Boswell et al. 2015). This highlights that treatment effectiveness is inherently multifaceted and may vary not only between individuals but also within individuals across dimensions.

Although some effects were evident for many patients, not all improved, and when aggregated on a group level, the effects were small and only reached significance for pain interference. This aligns with the broader literature on psychological treatments for chronic pain, where average effect sizes are typically small to moderate (Eccleston et al. 2013; Williams et al. 2020). Across patients, effects on depressive symptoms, pain interference and catastrophizing were most consistent. Overall, this pattern is in line with our previous randomized controlled efficacy trial (Boersma et al. 2019; Södermark et al. 2020). Overall treatment credibility and satisfaction were high, with treatment responders reporting slightly higher satisfaction. Treatment adherence and fidelity analyses revealed that while most patients stayed in treatment, non‐responders showed greater variability in session attendance and were less likely to complete later treatment phases involving exposure. Taken together, the results suggest that this treatment can be implemented in regular clinical care with some level of effectiveness but overall effects are modest and there is great variability across patients. The observed heterogeneity underscores the importance of ideographic outcome assessment and the added value of continuous evaluation of progression throughout the treatment (McAleavey et al. 2024; McCracken 2023).

At large, the treatment resulted in convincing effects for approximately one‐third of the patients included in the analysis. This was corroborated by significant changes on the standardized measures with large effect sizes. This can be compared to our efficacy randomized controlled trial, where 43% of participants showed clinically significant improvement on at least one of the outcomes (Boersma et al. 2019). While the criteria for robust treatment responses appear to give a valid estimate of who achieved a clinically relevant treatment effect, this suggests that there was only a modest proportion of patients with a robust treatment response. In addition, one‐fourth (*n* = 10) of the included patients (*N* = 41) had to be excluded from analyses due to lack of data. This could imply that the proportion with a clear treatment response is an overestimation. While reasons for non‐completion varied, and were not necessarily related to treatment, these findings call for caution when drawing conclusions about the implementation potential and treatment effectiveness.

While direct comparisons with our previous efficacy trial and other RCT's are inherently limited by differences in methodology, real‐world implementation often introduces more variability in procedures and outcomes. However, pragmatic trials are essential for exploring effectiveness in everyday clinical practice (Kazdin 2023). The modest proportion of patients with a robust treatment response may be related to a variety of aspects. First, it should be noted that patients were recruited from the flow of ordinary care. Many of these patients did not actively seek psychological treatment, which might have affected their engagement. Moreover, the current sample represents a clinical population with very high complexity. This is reflected in psychiatric comorbidity, health care utilization and sociodemographic factors such as unemployment, lower educational level and immigrant background.

However, noteworthy, our data showed that non‐responders had a lower proportion of treatment content dedicated to emotional or behavioural exposure—a core component of the intervention—and a substantial number of patients did not reach the exposure phases and thus received no exposure at all. Hence, treatment fidelity and progression through later treatment phases emerged as key factors associated with treatment success. Previous research suggests that both therapist factors, such as self‐efficacy to deliver exposure‐based interventions and patient factors, such as buy‐in to treatment or individual barriers, may influence the extent to which a treatment is utilized as intended (Aldadi et al. 2024; Goldsmith et al. 2023; Langthorne et al. 2023). The results from a complementary qualitative study with participating therapists, performed in parallel to this present study, indicate the potential relevance of therapist factors. Specifically, results revealed variability in the therapists' evaluation of the flexible format of the intervention which may have contributed to differences in treatment delivery across patients (Löfstrand et al. 2024). While designed to be adaptable, the treatment model was perceived as facilitating for some therapists, while others were struggling and would have preferred a higher degree of support. The lack of self‐efficacy experienced by some of the therapists may have contributed to differences in treatment delivery across patients. Indeed, exposure‐based techniques are well‐documented to be challenging for clinicians to implement consistently and prone to ‘therapist drift’ (Langthorne et al. 2023; Waller and Turner 2016).

At the same time, our data also suggests that lack of progression through treatment phases is not the sole explanation for the lack of an apparent effect. Importantly, the finding that some non‐responders completed treatment with full attendance and apparent fidelity and satisfaction indicates that other mechanisms—such as ineffective exposure, or mismatch between patient needs and treatment content or structure—could be at play. The variability in progression through treatment and its potential reasons is a subject in need of further study, with designs including larger sample sizes. However, taken together, the quantitative and qualitative findings from the present project point to the importance of training clinicians in chronic pain mechanisms and treatment techniques (Linton et al. 2024). In addition, methods such as ecological momentary assessment, single‐case experimental designs or network analyses may further advance our understanding of processes of change and its individual heterogeneity (Schemer et al. 2023; Scholten et al. 2024). In this light, the present study contributes to a more granular understanding of treatment response, extending prior group‐level findings.

### 4.1 Strengths and Limitations

The single‐case experimental design (SCED) allowed a detailed analysis of treatment effects on the individual level as well as aggregated inferences (Heyvaert and Onghena 2014; Van den Noortgate and Onghena 2008). The method is increasingly recognized as a viable and important addition to clinical intervention and effectiveness research (Nikles et al. 2021; Scholten et al. 2024). To evaluate treatment effectiveness, we used repeated outcome measurement alongside patient‐ and therapist evaluations. The identification of responders, defined as participants showing at least moderate effect from baseline through intervention and across outcomes, was strengthened by examining convergence with changes on standardized pre‐post measures. Still, treatment effects on the individual level were highly heterogeneous.

While the analytic N is sufficient for single case experimental design analyses, it is rather low for the subgroup analyses (specifically, the responder vs. non‐responder analyses), which intended to probe associations between treatment response and aspects of implementation. A larger sample would have enabled more robust subgroup‐level analyses. Another limitation is the lack of long‐term outcomes in terms of return to work and health care use, or health‐economic analyses. Although the convenience sampling procedure, based on clinician referrals, may have introduced selection bias and limited systematic control over eligibility, it also enabled recruitment of a clinically diverse sample across multiple care settings and geographical regions.

The real‐world implementation provides increased external validity (Kazdin 2023), but introduces variability in procedures. While we aimed to monitor this by process evaluation (Bernet et al. 2013; Curran et al. 2012), further investigation of patient‐, therapist‐factors and interaction during the course of treatment would provide additional insights into the relationship between implementation and treatment outcomes.

## 5 Conclusion

The findings support the feasibility and potential effectiveness of the hybrid emotion‐focused treatment for patients with comorbid chronic pain and emotional problems in routine clinical care. When performed by therapists in regular care, the treatment appears to benefit most patients to some extent and a smaller subset of patients to a clinically significant extent, particularly regarding pain interference in daily life and levels of emotional distress.

This treatment model is one of a range of new treatment approaches that aim to target pain–emotion connections explicitly and directly (Ashar et al. 2022; Lumley and Schubiner 2019; Michal et al. 2025; Norman‐Nott et al. 2022). These treatments vary in format and conceptual frame but are united in a focus on the patient's threat conceptions about pain and function while some also explicitly address emotions in a broader fashion. While these emotion‐focused treatments are topographically and conceptually different, they are united in their transdiagnostic focus on emotion regulation as a central psychological mechanism underlying the perpetuation of problems (Boersma and Flink 2025; Koechlin et al. 2018; Sloan et al. 2017).

The findings of this study further support a transdiagnostic approach for patients with comorbid chronic pain and emotional problems. Treatment effects varied across individuals, and treatment progression—specifically progression through exposure, which is a central part of the treatment—was linked to outcome. This highlights the importance of treatment fidelity and reinforces its value as a key factor to explore for successful further implementation.

## Author Contributions

Conceptualization: I.F., S.L. and K.B.; methodology, H.Z., X.Z., S.B., R.L., I.F., S.L. and K.B.; formal analysis, H.Z. and X.Z.; data curation, H.Z.; writing – original draft preparation, H.Z.; writing – review and editing, X.Z., S.B., R.L., I.F., S.L. and K.B.; project administration, R.L.; funding acquisition, K.B. All authors have read and agreed to the published version of the manuscript.

## Funding

This study was supported by AFA insurance (200042).

## Conflicts of Interest

The authors declare no conflicts of interest.

## Supporting information


**Figure S1:** Example graphs.
**Table S1:** Baseline characteristics.
**Table S2:** Treatment description.
**Appendix S1:** Standardized measures.
**Table S3:** Results standardized measures.
**Table S4:** Patient and therapist ratings of improvement.
