# Supplementary material for: Effectiveness of a Transdiagnostic Emotion‐Focused Treatment in Clinical Care: A Sequential Single‐Case Experimental Design
Source: Eur J Pain. 2026 Jun 13;30(6):e70310. doi: 10.1002/ejp.70310 (PMC13264282; doi:10.1002/ejp.70310)
Supplement: Supplementary file 1 — Figure S1: Example graphs. Table S1: Baseline characteristics. Table S2: Treatment description. Appendix S1: Standardized measures. Table S3: Results standardized measures. Table S4: Patient and therapist ratings of improvement. [file EJP-30-0-s001.docx]

**Supplemental material**

Supplemental material to *Effectiveness of a transdiagnostic emotion-focused treatment in clinical care: a sequential single-case experimental design (SCED)*. Zetterberg H, Zhao X, Bergbom S, Lennartsson R, Flink I, Linton S, Boersma K.

**Table S1.** Demographic and clinical characteristics at baseline among the n=10 participants that were lost to analysis and not included in the final sample.

|  | n=10 |
| --- | --- |
| Age, mean (SD) | 41.9 (10.9) |
| Gender, women, n (%) | 6 (60.0) |
| Nationality, Swedish, n (%) | 6 (60.0) |
| Education, n (%) |  |
| Middle school  Highschool/ vocational  University | 1 (10.0)  7 (70.0)  2 (20.0) |
| Occupational status, n (%) |  |
| Working  Unemployed  Student  Non-working (e.g. retirement pension) | 6 (60.0)  3 (30.0)  0 (0)  1 (10) |
| Sick leave past year, n (%) |  |
| 0-14 days  15-180 days  181-365 days | 2 (20.0)  3 (30.0)  5 (50.0) |
| Pain locations, n (%) |  |
| Back, neck, and/or shoulders  Legs and arms (including feet and hands)  Stomach  Head (e.g., headache, face, jaws, eyes)  Pain classification  Musculoskeletal pain  Widespread pain (spine and legs/arms)  Multisite symptom burden >3 sites  Number of pain locations*, median (IQR) | 8 (80.0)  9 (90.0)  6 (60.0)  6 (60.0)  10 (100.0)  7 (70.0)  7 (70.0)  6 (6) |
| Symptom duration > 1 year, n (%) | 10 (100.0) |
| Health care visits, median (range) past year |  |
| Physician  Specialist/hospital  Psychologist  Physiotherapist  Social worker  Other (e.g., chiropractor, acupuncturist)  Total number of visits >2, n (%) | 4 (0 - >10 visits)  0 (0 - >10 visits)  3 (0 - >10 visits)  5 (0 - >10 visits)  0 (0 - >10 visits)  1 (0 - >10 visits)  10 (100.0) |
| Screening measures, mean (SD) | n=4 |
| Function (OMPSQ, 0-40)  Pain interference (MPI, 0-6)  HADS anxiety (0-21)  HADS depression (0-21) | 26.3 (9.5)  5.0 (1.4)  13.0 (4.2)  15.5 (1.3) |
| Psychiatric comorbidity (MINI) n (%) | n=6 |
| Not fulfilling disorder criteria  Major depressive disorder  Anxiety disorder  Comorbid depressive and anxiety disorder | 0 (0)  5 (83.3)  5 (83.3)  4 (66.7) |

Notes: HADS, Hospital Anxiety and Depression Scale. MINI, Mini International Neuropsychiatric Interview 7.0.1. MPI, Multidimensional Pain Inventory item 2. OMPSQ, Orebro Musculoskeletal Pain Screening Questionnaire items 21-24. ^a^ Seven pain sites in total: neck, shoulders, upper back, lower back, arms/legs, stomach, and head.

**Table S2.** Overview of the hybrid emotion-focused treatment; stages and objectives.

| Treatment stage | | Objectives |
| --- | --- | --- |
| I. | **Building a working relationship, soothing distress, and developing relevant goals** | - Develop therapist-patient communication and trust (validation)  - Soothe negative emotions  Identify key emotions and pain and their triggers  - Develop personally relevant goals  - Introduce dialectics  - Analyze problem situations with chain analyses |
| II. | **Building skills to afford exposure** | - Normalize avoidance and underscore importance of exposure in didactic psychoeducation with validation  - Metaphors for associating exposure with goal pursuit - Finalize personally relevant goals as targets during treatment  - Develop a set of personalized skills for regulating pain and emotion  - Practice skills as preparation for exposure |
| III. | **Exposure for avoided movement and emotions** | - Exposure in-vivo for targeted avoidance of movements  - Exposure in-vivo for targeted avoidance of emotion  - Generalize with practice at home  - Apply toward goals and generalize via behavioral experiments |
| IV. | **Building skills for emotion-provoking social situations** | - Identify personally relevant social situations that trigger emotional distress related to the pain problem e.g. guilt, shame, or fear  - Build skills to deal with social interactions, especially those that provoke distress keying on communication skills e.g. validation and disclosure  - Improve interpersonal relationships by sharing intimate experiences, feelings, and pain (disclosure)  - Identify important cues for when, where, and with whom, one may disclose and share intimate feelings e.g. about pain or emotions  - Train how to disclose in a validating manner  - Apply emotion regulation skills to achieve disclosure and intimacy |
| V | **Continuing progress. Maintaining and refining** | - Psychoeducation about flare-ups and relapse (being prepared; never give up)  - Identify key skills  - Identify key stimuli that trigger avoidance |

Linton & Kleinberg (2020). *Treatment manual. Chronic pain and emotional distress*. Örebro University.

**Standardized measures**

**Appendix S1.**

The following standardized questionnaires were administered at baseline, post-intervention, and at 12-month follow-up:

Pain severity and pain interference were assessed by the Multidimensional Pain Inventory, Swedish version subscales ^1,2^. MPI-S is a well-used and validated measure. ^2^ An average score is calculated for each subscale (pain severity 2 items, pain interference 11 items), ranging 0-6. Cronbach’s alpha in the current study was 0.86 and 0.92, respectively.

Depressive symptoms were assessed by the self-rating version of the Montgomery Åsberg Depression Rating Scale, the MADRS-S which has acceptable reliability and validity. ^3,4^ The MADRS-S has nine items rated 0-6. The total score ranges 0-54 and is interpreted: 0-12 = no depression, 13-19 = mild depression, 20-34 = moderate depression, >35 = severe depression. Cronbach’s alpha in the current study was 0.77.

Emotion regulation was assessed by the Difficulties in Emotion Regulation Scale, 16 item version, which has shown good reliability and validity. ^5^ DERS-16 assess the following domains: nonacceptance of negative emotions, inability to engage in goal-directed behaviors when distressed, difficulties controlling impulsive behaviors when distressed, limited access to emotion regulation strategies perceived as effective, and lack of emotional clarity. A total score is calculated, ranging 16-80 with higher scores reflecting greater difficulties. Cronbach’s alpha in the current study was 0.95.

Avoidance and cognitive fusion were assessed by the Psychological Inflexibility in Pain questionnaire. ^6^ The PIPS is a 12-item scale designed to measure psychological inflexibility in patients with pain and has shown acceptable internal consistency and validity. ^6^ A total score is calculated for each subscale, ranging 10-70 for avoidance and 6-42 for cognitive fusion, lower scores being beneficial. Cronbach’s alpha in the current study were 0.84 and 0.61, respectively.

Disability at work was assessed by the Work Limitations Questionnaire -16 (WLQ-16), which has shown promising psychometric properties. ^7^ In the WLQ, the impact of health problems on occupational performance is measured. An index scale 0-100 is calculated, where higher scores indicate more problems. Cronbach’s alpha in the current study was 0.90.

Sleep problems were assessed by the Insomnia Severity Index (ISI), which is a valid and reliable measure of clinical insomnia. ^8^ Seven items cover severity of insomnia, dissatisfaction with sleep, interference with daily functioning, noticeability of impairment, and distress related to the sleep problem. The total score ranges 0-28 and is interpreted: <8 = no insomnia, 8-14 = sub-threshold insomnia, 15-21 = clinical insomnia (moderate severity), and 22-28 = severe clinical insomnia. Cronbach’s alpha in the current study was 0.88.

Perceived health was measured using a visual analogue scale (VAS) ^9^ where participants rated perceived health during the last 30 days. The scale was in digital format, horizontal and anchored with 0=worst imaginable and 100=best imaginable.

### **References**

1. Kerns RD, Turk DC, Rudy TE. The West Haven-Yale Multidimensional Pain Inventory (WHYMPI): *Pain*. 1985;23(4):345-356. doi:10.1016/0304-3959(85)90004-1

2. Bergström G, Jensen IB, Bodin L, Linton SJ, Nygren ÅL, Carlsson SG. Reliability and factor structure of the Multidimensional Pain Inventory – Swedish Language Version (MPI-S): *Pain*. 1998;75(1):101-110. doi:10.1016/S0304-3959(97)00210-8

3. Cunningham JL, Wernroth L, Von Knorring L, Berglund L, Ekselius L. Agreement between physicians’ and patients’ ratings on the Montgomery–Åsberg Depression Rating Scale. *J Affect Disord*. 2011;135(1-3):148-153. doi:10.1016/j.jad.2011.07.005

4. Fantino B, Moore N. The self-reported Montgomery-Åsberg depression rating scale is a useful evaluative tool in major depressive disorder. *BMC Psychiatry*. 2009;9(1):26. doi:10.1186/1471-244X-9-26

5. Bjureberg J, Ljótsson B, Tull MT, et al. Development and Validation of a Brief Version of the Difficulties in Emotion Regulation Scale: The DERS-16. *J Psychopathol Behav Assess*. 2016;38(2):284-296. doi:10.1007/s10862-015-9514-x

6. Wicksell RK, Lekander M, Sorjonen K, Olsson GL. The Psychological Inflexibility in Pain Scale (PIPS) – Statistical properties and model fit of an instrument to assess change processes in pain related disability. *Eur J Pain*. 2010;14(7). doi:10.1016/j.ejpain.2009.11.015

7. Beaton DE, Kennedy CA. Beyond return to work: testing a measure of at-work disability in workers with musculoskeletal pain. *Qual Life Res*. 2005;14(8):1869-1879.

8. Bastien C. Validation of the Insomnia Severity Index as an outcome measure for insomnia research. *Sleep Med*. 2001;2(4):297-307. doi:10.1016/S1389-9457(00)00065-4

9. Feng Y, Parkin D, Devlin NJ. Assessing the performance of the EQ-VAS in the NHS PROMs programme. *Qual Life Res*. 2014;23(3):977-989.

**Results standardized measures**

**Table S3.** Self-report measures at pre-treatment, post-treatment, and 12-months follow-up organized according to treatment responders (n=10) and non-responders (n=21).

| Outcome variable,  mean (SD) | Treatment responders n=10 | Within-group statistics | Effect size, *d* | Non-responders n=21 | Within-group statistics | Effect size, *d* |
| --- | --- | --- | --- | --- | --- | --- |
| Pain severity (MPI) |  |  |  |  |  |  |
| Pre | 4.75 (1.01) |  |  | 4.31 (1.01) |  |  |
| Post | 3.70 (1.36) | t(9) =2.11, p=0.06 | 0.67 | 4.50 (1.07) | t(18) =-1.14, p=0.26 | -0.26 |
| 12-months | 3.08 (1.11) | t(5) =7.27, p<0.001* | 2.97 | 4.42 (1.36) | t(11) =-0.10, p=0.93 | -0.03 |
| Pain interference (MPI) |  |  |  |  |  |  |
| Pre | 5.16 (1.15) |  |  | 4.84 (0.96) |  |  |
| Post | 4.18 (1.47) | t(9) =3.30, p=0.009* | 1.04 | 4.67 (1.07) | t(18) =1.36, p=0.19 | 0.31 |
| 12-months | 3.56 (1.16) | t(5) =5.42, p=0.003* | 2.21 | 4.88 (1.41) | t(11) =-0.07, p=0.95 | -0.03 |
| Depression  (MADRS-S) |  |  |  |  |  |  |
| Pre | 31.30 (7.54) |  |  | 26.52 (7.01) |  |  |
| Post | 22.50 (11.23) | t(9) =3.41, p=0.008* | 1.08 | 27.17 (8.85) | t(17) =-0.48, p=0.64 | -0.11 |
| 12-months | 21.67 (12.27) | t(5) =2.68, p=0.044* | 1.10 | 27.50 (9.39) | t(11) =0.26, p=0.80 | 0.08 |
| Avoidance (PIPS) |  |  |  |  |  |  |
| Pre | 47.10 (7.67) |  |  | 42.76 (7.34) |  |  |
| Post | 38.00 (12.27) | t(9) =3.35, p=0.009* | 1.06 | 39.39 (10.31) | t(17) =2.38, p=0.03 | 0.56 |
| 12-months | 36.67 (11.91) | t(5) =2.78, p=0.039* | 1.14 | 40.92 (9.89) | t(11) =1.49, p=0.17 | 0.43 |
| Fusion (PIPS) |  |  |  |  |  |  |
| Pre | 23.90 (3.03) |  |  | 21.81 (3.70) |  |  |
| Post | 19.70 (5.70) | t(9) =2.60, p=0.03* | 0.82 | 20.89 (5.52) | t(17) =0.72, p=0.48 | 0.17 |
| 12-months | 17.83 (4.45) | t(5) =3.39, p=0.019* | 1.38 | 21.00 (3.98) | t(11) =1.72, p=0.11 | 0.50 |
| Emotion regulation (DERS) |  |  |  |  |  |  |
| Pre | 54.80 (11.13) |  |  | 41.86 (16.26) |  |  |
| Post | 44.50 (15.43) | t(9) =2.91, p=0.017* | 0.92 | 44.00 (17.75) | t(17) =-1.56, p=0.14 | -0.37 |
| 12-months | 49.50 (13.61) | t(5) =3.16, p=0.025* | 1.29 | 50.08 (18.11) | t(11) =-1.73, p=0.11 | -0.50 |
| Work limitations (WLQ-16) |  |  |  |  |  |  |
| Pre | 73.13 (19.76) |  |  | 57.60 (14.50) |  |  |
| Post | 57.44 (25.43) | t(9) =1.59, p=0.15 | 0.53 | 58.25 (23.16) | t(16) =-0.31, p=0.76 | -0.08 |
| 12-months | 55.04 (27.14) | t(4) =1.72, p=0.16 | 0.77 | 70.44 (23.15) | t(9) =-1.31, p=0.22 | -0.41 |
| Insomnia (ISI) |  |  |  |  |  |  |
| Pre | 18.50 (6.52) |  |  | 15.76 (6.77) |  |  |
| Post | 14.10 (5.80) | t(9) =4.54, p=0.001* | 1.44 | 15.11 (6.42) | t(17) =-0.09, p=0.93 | -0.02 |
| 12-months | 15.80 (5.02) | t(4) =2.44, p=0.07 | 1.09 | 16.67 (7.22) | t(11) =-1.08, p=0.30 | -0.31 |
| Health (VAS) |  |  |  |  |  |  |
| Pre | 24.90 (13.89) |  |  | 33.10 (17.83) |  |  |
| Post | 44.30 (14.68) | t(9) =-3.94, p=0.003* | -1.25 | 31.94 (21.58) | t(17) =0.41, p=0.67 | 0.10 |
| 12-months | 52.40 (22.47) | t(5) =-2.97, p=0.041* | -1.33 | 30.67 (23.14) | t(11) =-0.40, p=0.70 | -0.12 |

Notes: Within groups statistics are paired t-test. Effect size Cohen’s *d*; 0.2 = small, 0.5 = medium, 0.8 = large, 1.2 = very large. *p-value significant at <0.05

DERS-16, The Difficulties in Emotion Regulation Scale (16-80). ISI, Insomnia Severity Index (0-28). MADRS-S, Montgomery Åsberg Depression Rating Scale (0-54). MPI, Multidimensional Pain Inventory (0-6), Psychological Inflexibility in Pain Scale avoidance subscale (10-70) and fusion subscale (6-42). SD, Standard deviation. VAS-health, visual analogue scale (0-100). WLQ-16, Work Limitations Questionnaire -16 item (0-100).

Missing data: Treatment responders: pre-treatment n=10 , post-treatment n=10 except WLQ n=9, 12-months follow-up n=6 except WLQ, ISI, EQ-VAS n=5 Non-responders: pre-treatment n=21 except WLQ n=20, post-treatment n=19 except MADRSS, PIPS, ISI, EQ-VAS all n=18 and WLQ n=17, 12-months follow-up n=12 except WLQ n=10

**Table S4.** Cross-tabulation of patient and therapist ratings of improvement.

| **Therapist ratings** | **Patient ratings** | |
| --- | --- | --- |
|  | Improved | Not improved |
| Improved | 7 | 3 |
| Not improved | 3 | 6 |

Notes:

Patient rating: Dichotomized variable from global improvement after treatment.

Therapist rating: Dichotomized variable from goal achievement.

**Example graphs**


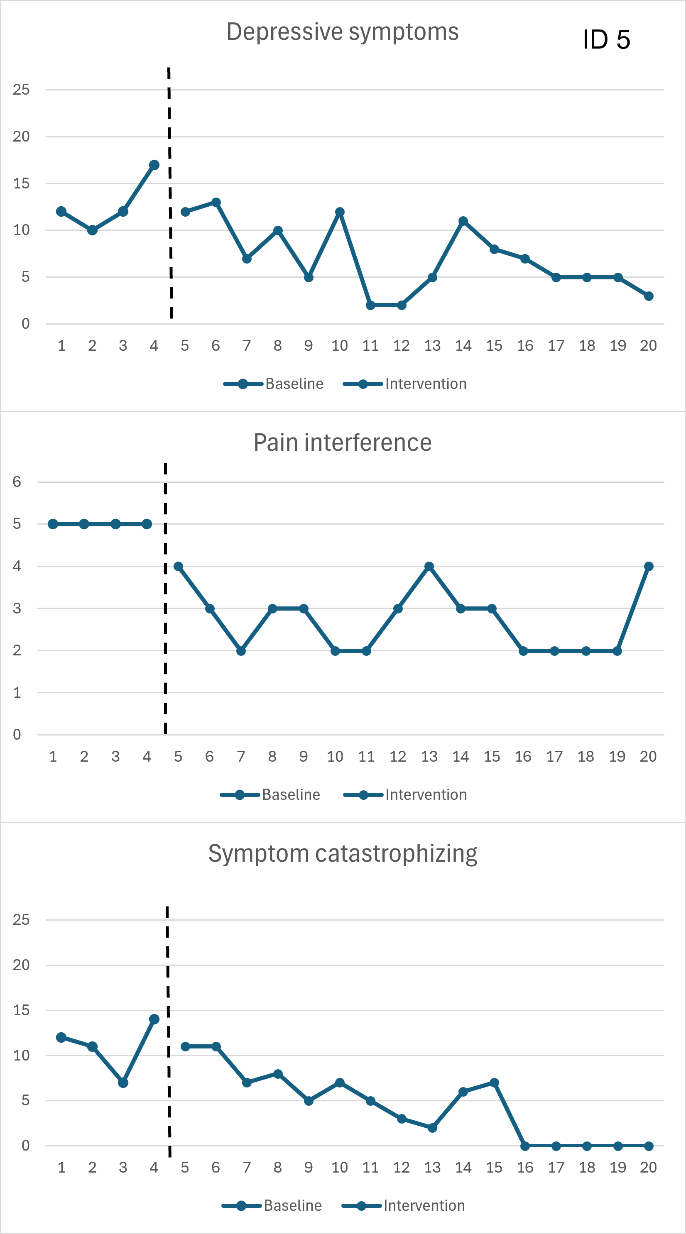

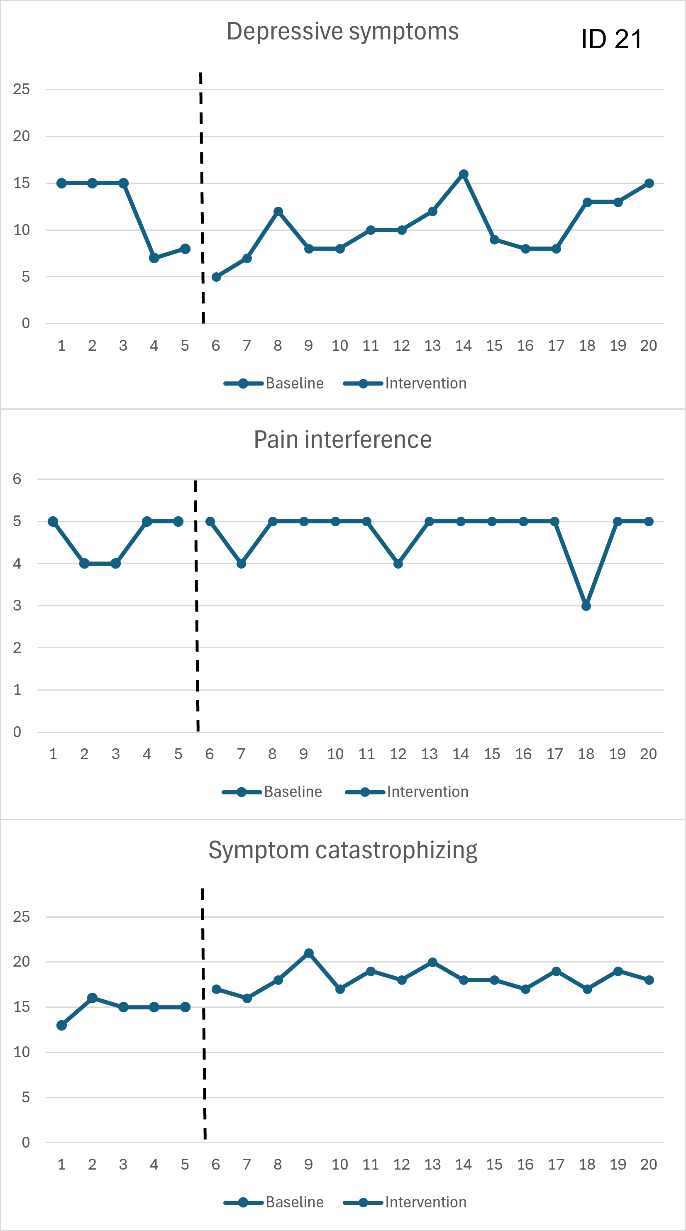


**Figure S1.** Example graphs of a treatment responder (ID 5) to the left and a non-responder (ID 21) to the right.

Notes: Depressive symptoms: Patient Health Questionnaire 9-item depression module (PHQ-9), range 0-27. Pain interference: Single item from the Multidimensional Pain Inventory (MPI-S), range 0-6.
Symptom catastrophizing: Symptom catastrophizing scale (SCS), range 0-28.
